# Pressure Orientation-Dependent Recovery of 3D-Printed PLA Objects with Varying Infill Degree

**DOI:** 10.3390/polym13081275

**Published:** 2021-04-14

**Authors:** Guido Ehrmann, Andrea Ehrmann

**Affiliations:** 1 Virtual Institute of Applied Research on Advanced Materials (VIARAM); guido.ehrmann@gmx.de; 2 Faculty of Engineering and Mathematics, Bielefeld University of Applied Sciences, 33619 Bielefeld, Germany

**Keywords:** polylactic acid (PLA), fused deposition modeling (FDM), three-point bending test, infill parameters, infill density, shape-memory properties

## Abstract

Poly(lactic acid) is not only one of the most often used materials for 3D printing via fused deposition modeling (FDM), but also a shape-memory polymer. This means that objects printed from PLA can, to a certain extent, be deformed and regenerate their original shape automatically when they are heated to a moderate temperature of about 60–100 °C. It is important to note that pure PLA cannot restore broken bonds, so that it is necessary to find structures which can take up large forces by deformation without full breaks. Here we report on the continuation of previous tests on 3D-printed cubes with different infill patterns and degrees, now investigating the influence of the orientation of the applied pressure on the recovery properties. We find that for the applied gyroid pattern, indentation on the front parallel to the layers gives the worst recovery due to nearly full layer separation, while indentation on the front perpendicular to the layers or diagonal gives significantly better results. Pressing from the top, either diagonal or parallel to an edge, interestingly leads to a different residual strain than pressing from front, with indentation on top always firstly leading to an expansion towards the indenter after the first few quasi-static load tests. To quantitatively evaluate these results, new measures are suggested which could be adopted by other groups working on shape-memory polymers.

## 1. Introduction

3D printing is nowadays no longer only used for rapid prototyping, but due to the emergence of new and improved techniques, as well as new materials, is also suitable for rapid production. Especially for single objects, preparing a mold for vacuum casting or similar techniques would be much too time and cost consuming [1]. This is one of the reasons why, e.g., biotechnological or biomedical solutions for single patients can often be 3D printed [2,3,4]. Additionally, 3D printing offers the possibility of designing patient-specific tissues with a sufficiently high printing resolution [5,6].

On the other hand, the most frequently used 3D printing materials for the fused deposition modeling (FDM) technique, i.e., acrylonitrile butadiene styrene (ABS), poly(lactic acid) (PLA) and a few others, often cannot reach the desired mechanical properties and the low surface roughness and waviness which are necessary in some applications [7,8,9]. Typically, the reduced mechanical properties are counteracted by heat post-treatments, by integrating nanofibers or nanoparticles [10,11,12] or continuous fibers [13,14].

Besides the good printability, PLA has other interesting characteristics, i.e., shape memory properties. This means that a 3D-printed object from PLA can be deformed to a certain degree and subsequently regenerated into its original shape through heat treatment, typically in the temperature range of 60–100 °C [15,16,17,18]. While other 3D printing polymers, such as poly(vinyl alcohol), poly(ε-caprolactone) or polyurethane, also show shape-memory properties [19,20,21,22], PLA is often advantageous due to its ease of printing, the relatively low price, and especially its biocompatibility.

The greatest problem in using PLA printed objects for shape-memory applications is the aforementioned danger of broken areas. In a previous study, we investigated the influence of the infill pattern and degree on the mechanical properties of 3D-printed cubes under a quasi-static load [23,24] and found the so-called gyroid infill to show promising recovery properties. The impact of the infill properties on the flexural and tensile properties of 3D-printed samples was evaluated in diverse previous studies, showing the importance of choosing them carefully to gain a desired response to mechanical deformation [25,26,27].

Here, we investigate the influence of the orientation of the applied pressure with respect to the sample axes and suggest several methods to quantitatively evaluate the recovery process. Opposite to most of the aforementioned studies, here, deformation is applied without heating the samples so that breaks can more easily occur. It should be mentioned that preliminary tests with soft PLA did not reveal a shape memory effect, so this material is not suitable for this purpose. This study aims at identifying possible new structures for protecting people during accidents, but that can also be applied to orthoses, bumpers and other structures that are prone to erroneous overloads, and could give way to the applied pressure and afterwards be restored again. Knowing the influence of the impact direction on the resulting residual deformation and the ability to recover the original shape is important for tailoring 3D-printed bumpers, orthoses, etc., in such a way that the best recovery is enabled due to the smallest amount of broken structures.

Additionally, different new measures are suggested to quantitatively describe the efficiency of the recovery process so that a rating of the infill degrees and pressure directions becomes possible.

## 2. Materials and Methods

The samples used in this study were printed with an FDM 3D printer I3 MK3 (Prusa Research A.S., Prague, Czech Republic). Corresponding to the nozzle diameter of 0.4 mm, a layer thickness of 0.2 mm for the first layer and 0.15 mm for all other layers was chosen, respectively. Typical printing temperatures (215 °C for the first layer, 210 °C thereafter) and heating bed temperatures (60 °C) were maintained during printing the objects with dimensions of (20 mm)^3^. The PLA used in this investigation was purchased from Prusa; an FTIR graph is shown in a previous paper [28].

**Figure 1:**
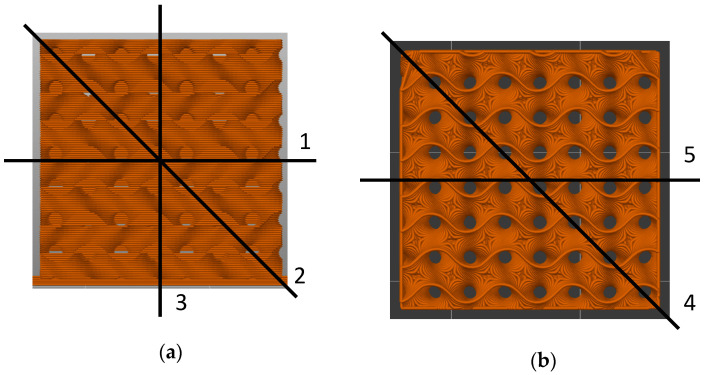
Front (**a**) and top view (**b**) on the gyroid sample with 20% infill. The lines along which a pressure was applied are defined as follows: (1) front horizontal, (2) front diagonal), (3) front vertical), (4) top diagonal, (5) top parallel to the front edge.

Based on the previous studies [23,24], the infill pattern “gyroid” was chosen, using 15%, 20%, and 25% infill degree, here denoted as G15, G20 and G25. All six walls are open, i.e., the numbers of perimeters and top/bottom layers is 0. For the lowest infill degree, this pattern is depicted in Figure 1. As visible here, this pattern has pores penetrating through the whole sample, in this way enabling regenerating the samples in a hot water bath. The lines along which a pressure was applied during the experiments are defined in Figure 1. These lines were chosen to evaluate all easily accessible orientation for a future application of such systems, e.g., in car bumpers or other objects made to protect people or objects during accidents.

To evaluate the recovery properties of these samples after application of a quasi-static load, a universal testing machine (Kern & Sohn GmbH, Balingen-Frommern, Germany) was used with a three-point bending test in which the doubled load pins on the lower side are exchanged by an even area. This means that the single pin of the three-point bending test was pressed into the specimen under examination, testing a local pressure instead of a global one and thus focusing on possibly broken bonds between neighboring areas with and without applied force. The tests were stopped after an impression of 10 mm, i.e., half of the sample dimension; alternatively, after a force of 1700 N was reached, to avoid approaching the maximum possible force of 2 kN, which can be measured with the used load cell. The test speed was 6 mm/min, 10 tests were performed per sample, typically with one repetition of the test, in some cases (tests with the force applied horizontally on the front) with two repetitions. Since no significant differences occurred between the repetitions, even the nearly complete breaking of the samples with the force applied horizontally on the front varying by max. one cycle, the replicates are now shown here.

To recover the samples, they were introduced into a water bath with a temperature of (60 ± 1) °C for 1 min, followed by cooling them down in a water bath at room temperature for 1 min. The recovery temperature and duration are known to strongly influence the process [18]; however, optimization of these parameters was not in the focus of the recent study. Instead, the effect of the applied pressure on breaking of different areas was investigated to optimize the orientation of the examined structure with respect to an external load.

Optical investigation of the samples was performed with a digital microscope Camcolms2 (Velleman, Gavere, Belgium).

## 3. Results and Discussion

**Figure 2:**
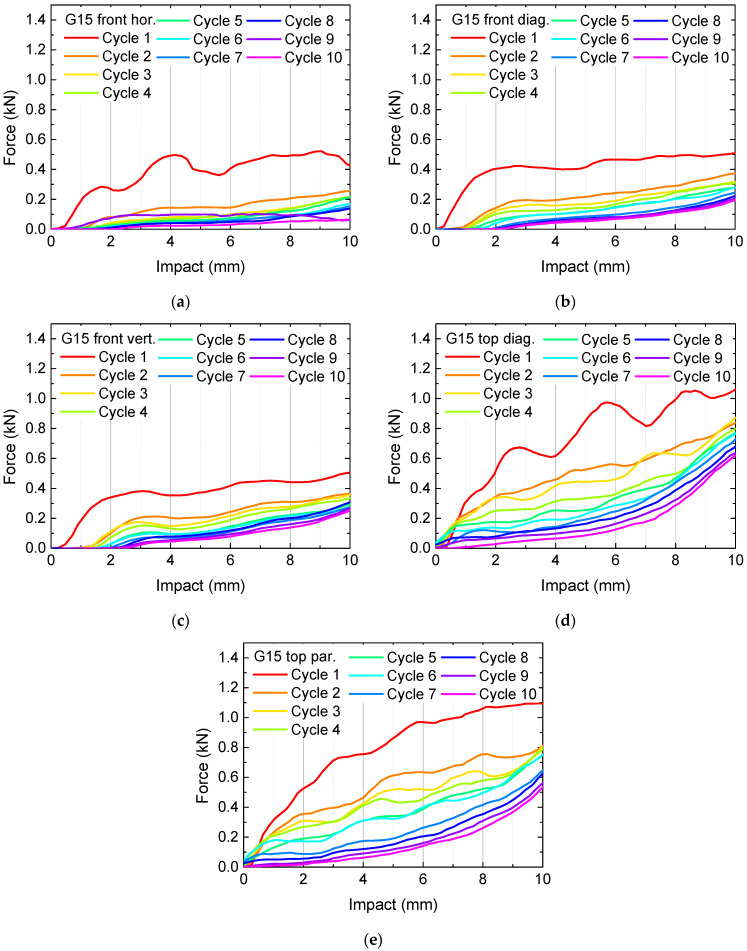
Quasi-static load tests in original state and after several test and recovery processes of samples G15: (**a**) front horizontal; (**b**) front diagonal; (**c**) front vertical; (**d**) top diagonal; (**e**) top parallel to the edges.

The results of the quasi-static load tests performed on samples G15 are depicted in Figure 2. The measured forces are given as a function of the impact.

Generally, there is always a large difference between the first curve (red) and the subsequent ones visible. Especially for the measurements with an impact applied horizontally on the front (Figure 2a) and diagonally on top (Figure 2d), the first curves show large irregular oscillations, corresponding to breaking of large areas, as can clearly be heard during the test.

**Figure 3:**
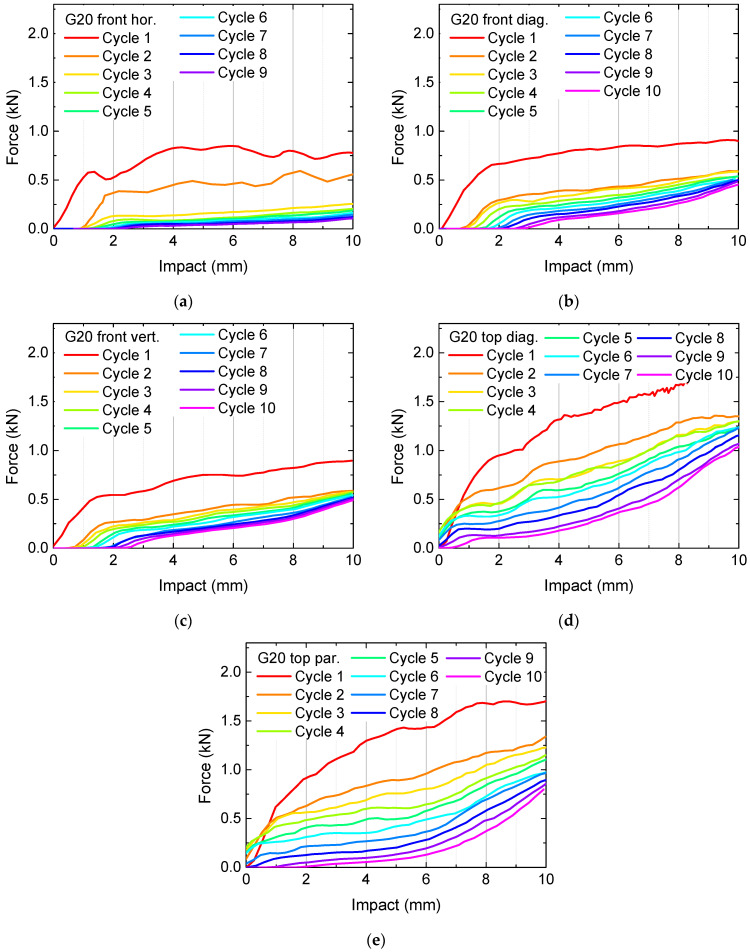
Quasi-static load tests in original state and after several test and recovery processes of samples G20: (**a**) front horizontal; (**b**) front diagonal; (**c**) front vertical; (**d**) top diagonal; (**e**) top parallel to the edges. The y-axes are scaled differently from Figure 2.

**Figure 4:**
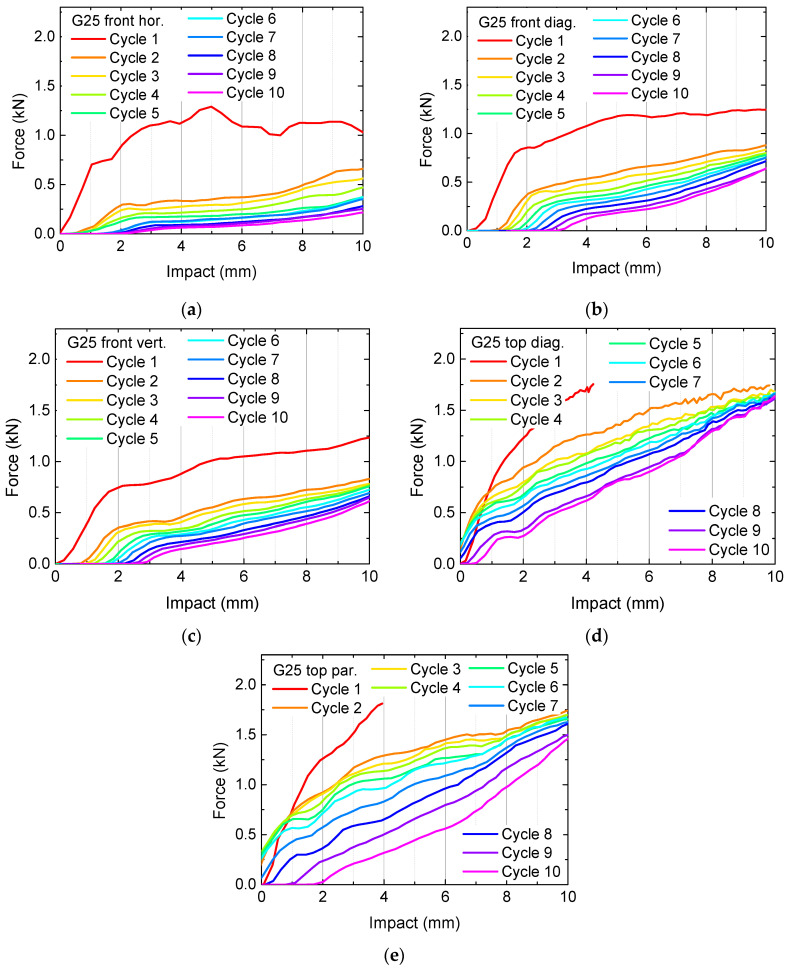
Quasi-static load tests in original state and after several test and recovery processes of samples G25: (**a**) front horizontal; (**b**) front diagonal; (**c**) front vertical; (**d**) top diagonal; (**e**) top parallel to the edges. The y-axes are scaled identically to Figure 3. In (**d**,**e**), the red lines are broken around 4 mm since to force limit was reached.

Besides these findings, the forces necessary for the applied deformation are not very high, suggesting using higher infill degrees. The corresponding results are depicted in Figure 3 for the samples G20 and in Figure 4 for the samples G25. In Figure 3a, cycle 10 is missing, since the sample broke nearly fully after nine test cycles.

Generally, the necessary forces for identical impacts increase with increasing infill degree, as expected. Horizontally pressing on the front, i.e., parallel to the printed layers, results in relatively small forces already for the first cycle and fast destroying of the samples, visible in the significantly reduced forces after the first or second test cycle. This finding can be explained by the smaller adhesion between adjacent layers in comparison with the adhesion within one layer, making this impact orientation (Figure 2a, Figure 3a and Figure 4a) especially susceptible for delamination and subsequent full splitting of the samples.

Another general finding is that for a pressure on top of the samples, there are always higher forces necessary for reaching a certain impact depth, as compared to an impact on the front. This property is apparently valid for the gyroid infill pattern in general; however, other patterns may lead to different orientations being able to take up the highest forces. Additionally, this property is not the most important one, since the previous results clearly show that higher forces can be reached by higher infill degrees. Instead, it is necessary to examine the recovery properties.

Since these properties cannot be directly estimated from the previous graphs, here we suggest a few different calculations which enable a quantitative evaluation of the recovery of the samples under examination. Firstly, it can be useful to measure a force ratio, e.g., related to a force found in the first cycle, and calculate the forces necessary to reach the identical impact after 5 or 10 test cycles. For a perfect recovery process, values of 1 could be expected.

**Figure 5:**
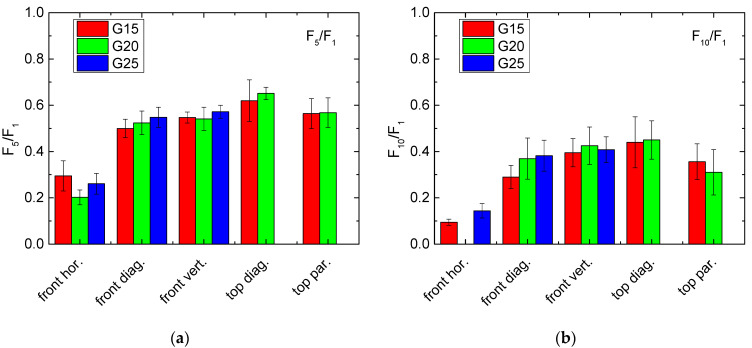
Force quotients of the samples under investigation, related to the force measured along an impact of 8–10 mm during the first cycle: (**a**) F_5_/F_1_; (**b**) F_10_/F_1_.

This definition is, however, not really suitable, as the often large oscillations in the curves in Figure 2, Figure 3 and Figure 4 show, especially for the first cycle on which all calculations should be based. Thus, it is necessary to define a certain impact range instead of a single value for which an average force is calculated. Here, we suggest using the last 20% of the whole range of impact values, i.e., the forces calculated in the impact range from 8 mm to 10 mm. Figure 5 depicts the results of these evaluations. The missing data are related to unfinished first cycles (in case of sample G25, missing blue bars) and to a missing 10th cycle (in case of G20 front horizontal).

Despite these few missing values, there is a clear trend visible that “front horizontal” shows the lowest recovery properties, as already seen in the previous figures. On the other hand, a slight trend towards higher recovery properties for pressing on top in only visible for the quotient F_5_/F_1_, while for F_10_/F_1_, the measurement on top parallel to one of the edges shows slightly smaller values than those gained by pressing on the front diagonally or vertically. Generally, as clearly visible by the error bars, these deviations are not significant.

**Figure 6:**
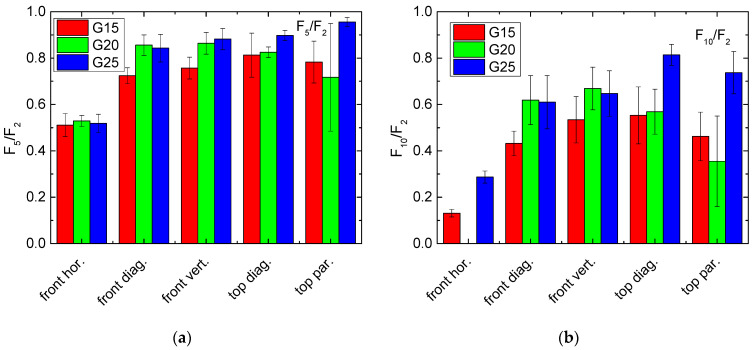
Force quotients of the samples under investigation, related to the force measured along an impact of 8–10 mm during the second cycle: (**a**) F_5_/F_2_; (**b**) F_10_/F_2_.

Another possibility for defining a quotient giving rise to the recovery properties of the samples under examination is given by using the second cycle as the reference. This would have the advantage that the missing values due to too high forces during the first cycle can be added. Additionally, the large difference between the first and the second cycle that was visible for all samples would be ignored, so that quotients related to the second cycle may be regarded to be more related to the long-term properties, without overemphasizing the first cycle with its special properties. The results of these evaluations are presented in Figure 6, again for averaging over the range between 8 mm and 10 mm impact.

Here, the differences between the fifth and the second cycle are clearly reduced and generally near to the desired value of 1, as well as pressing horizontally on the front (Figure 6a). On the other hand, comparing the forces necessary during the tenth and the second cycle (Figure 6b), a clear advantage of the values for G25, pressed from top, is visible. It must be mentioned, however, that the differences are again not significant, as visible by the error bars.

**Figure 7:**
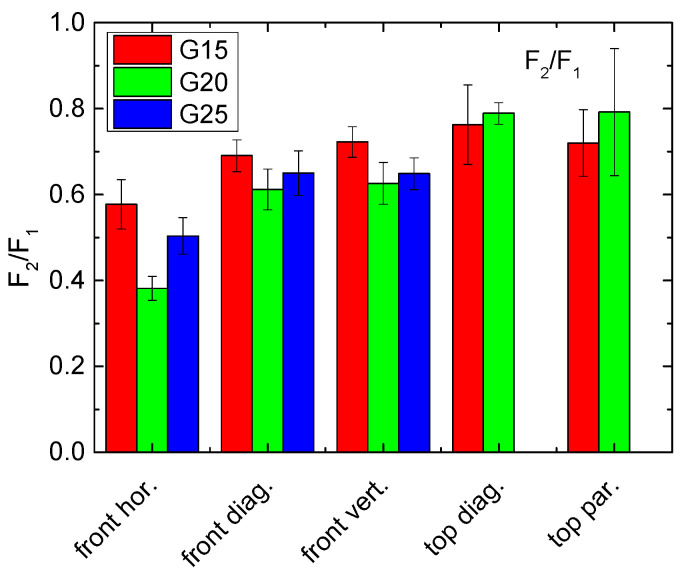
Force quotients of the samples under investigation, calculated from the forces measured along an impact of 8–10 mm during the second and the first cycle F_2_/F_1_.

As a third possibility for building force gradients, Figure 7 shows the ratio of the forces during the second and the first cycle, again measured for an impact of 8–10 mm. While an impact horizontally along the front is again slightly less recovered, there are no clear differences visible between the other results.

Comparing these evaluations, it can be stated that F_10_/F_2_ may be advantageous since it shows relatively strong differences, and the problem of a potentially too high force during the first cycle can be overcome. On the other hand, the evaluation method must always correspond to the desired application, especially to the question whether recovery will only be performed once, e.g., to reduce the consequences of a traffic accident so that a vehicle can be driven to the workshop where it is repaired, or whether an object will be exposed to steady small deformations which it should withstand for long times.

**Figure 8:**
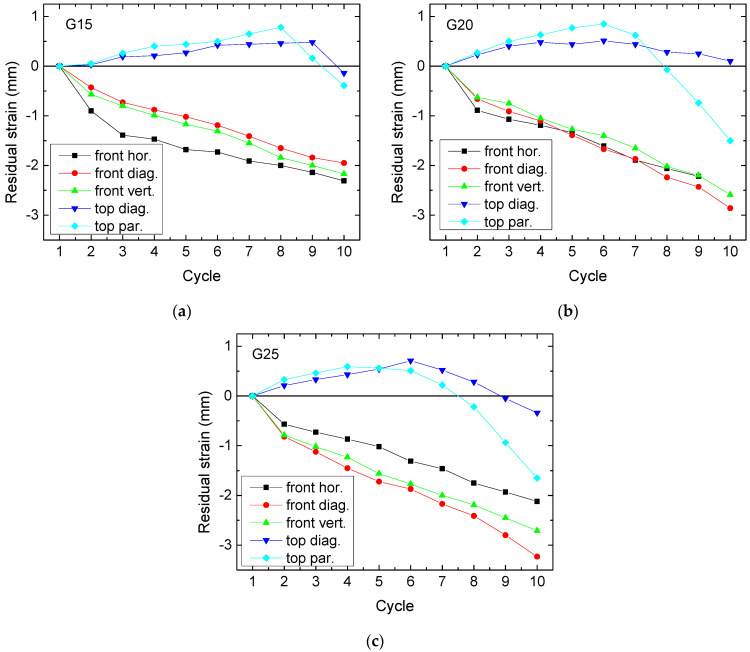
Cycle-dependent residual strain, measured for different sets of samples: (**a**) G15; (**b**) G20; (**c**) G25.

After these force-related evaluations, another value should be mentioned that gives rise to another property related to the recovery process, i.e., the residual strain. It is well known that continuous deformation of elastic polymers may lead to such a residual strain, i.e., a deformation left when no forces act on the specimen anymore [29]. Here, it could be expected that similarly a negative residual strain remains after each test due to incomplete recovery. As Figure 8 shows, this is not always the case.

While pressing on the front indeed leads to the expected negative residual strain, i.e., a durable compression due to incomplete recovery, pressing on top always leads to a positive residual strain during the first cycles. This means that the samples expand firstly into the direction of the applied force. Indeed, this behavior can be observed during testing. It is connected first with broken bonds along the area on which the pressure is applied, leading to small parts protruding from the main block. This effect was already observed in the previous study [23,24], but not quantified there.

For a comparison of the curves in Figure 8, it should be noted that the ideal curve would stay constant around 0 mm or at least have a small negative gradient. Interestingly, this is mostly the case for the smallest infill degree, i.e., samples G15, pressed from the front. Pressing on top may, at first glance, look beneficial; however, in all cases a maximum is visible between the first increasing values and the subsequent decreasing ones which is in most cases followed by a sharp decline of the curves for pressing parallel to the edges. Only pressing diagonally from the top results in a smaller negative slope or values similar to pressing on the front.

**Figure 9:**
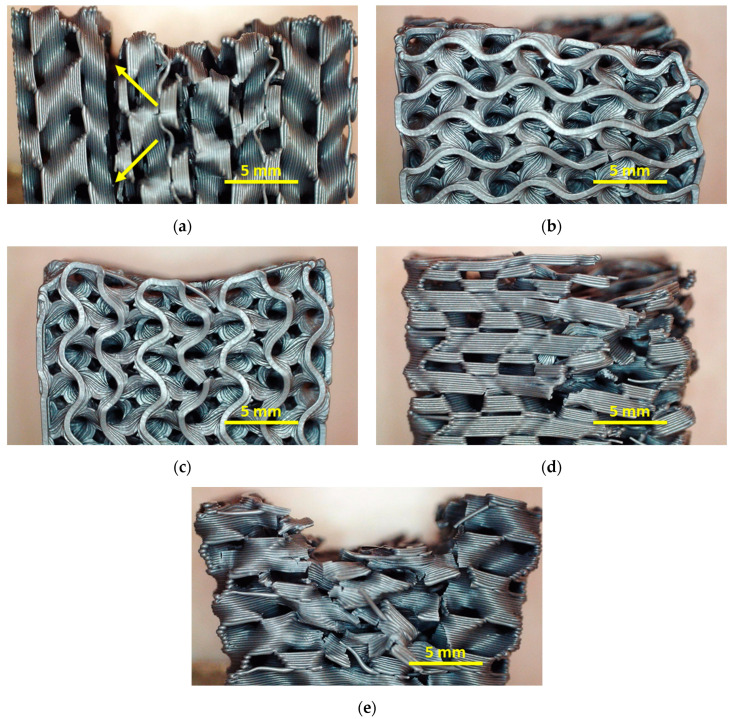
Microscopic images of samples after 10 pressure and recovery cycles: (**a**) front horizontal with full break (between arrows); (**b**) front diagonal, image taken from top; (**c**) front vertical; (**d**) top diagonal; (**e**) top parallel.

To enable a better understanding of the effects due to pressing along the different orientations, Figure 9 depicts images of some samples after the 10th testing and recovery cycle. Since there are no qualitative differences visible between the samples with different infill degree, here we show the average infill degree of 20%.

Comparing these images, it is firstly visible that strong deformations occur for pressing horizontally onto the front, as already concluded from the previous quantitative examinations (Figure 9a). It is also clearly visible that the sample pressed horizontally onto the front has become significantly “broader”, as compared to the other samples (all images taken with identical magnification).

The largest deformation is visible for pressing on top parallel to one of the edges (Figure 9e). At first glance, this does not seem to be consistent with the measurement of the residual strain shown in Figure 8b; however, the latter does not measure the residual deformation along the pressure line, but the maximum height. Here, several broken strands are visible, explaining the fast decrease of the residual strain visible in Figure 8b. It can be expected that within a few further test cycles, the sample will be distorted much more strongly, or even fully destroyed.

Interestingly, this is different for pressing vertically on the front (Figure 9c). While the forces necessary for the desired impact are significantly lower in this case as during pressing on top, here a relatively small deformation and only few broken strands are visible, making this orientation a potential candidate for future applications.

Finally, both diagonal pressing orientations (Figure 9b,d) show a clear residual deformation, in the case of pressing diagonally on the top of the sample with several broken strands, which is again not the case for pressing on the front. This finding again shows that, depending on the application and the expected forces, pressing on the front may be more suitable due to a smaller amount of broken bonds and thus higher recovery ability.

Concluding these different quantitative and qualitative evaluations, it can be stated that pressing horizontally on the front generally leads to insufficient results. 

For pressing on top, the diagonal seems to be advantageous; however, it must be tested in the next study whether this changes with the orientation of the infill and whether a 45° orientation of the gyroid infill (cf. Figure 1b) supports recovery. No clear differences are visible between the different orientations used to press on the front in terms of the residual strain; for the highest infill degree, the geometrical recovery (i.e., the smallest residual strain) is even found for pressing horizontally on the front, the situation found most unstable in terms of force gradients.

Regarding the force gradients depicted in Figure 5, Figure 6 and Figure 7, only G25 pressed on top shows a slight advantage. Combined with the residual strain evaluation, G25 pressed from the top diagonally can be suggested as a preliminary favorite.

However, more examinations are necessary, possibly including more evaluation methods, to investigate whether this sample offers the optimum recovery properties in case of 3D printing with pure PLA.

## 4. Conclusions and Outlook

Square specimens 3D-printed from the shape-memory polymer PLA were subjected to repeated pressure tests, using a modified three-point bending test. After a previous study revealed that the gyroid infill pattern is suitable for a relatively small amount of broken bonds during mechanical impact, here the influence of the impact orientation with respect to the cube axes was examined. Generally, pressing from the top allowed higher forces to be taken up for identical impacts. Pressing horizontally on the front, i.e., parallel to the printed layers, resulted in all cases in partial or full destruction of the sample. Combining quantitative evaluations by different force quotients with measurements of the residual strain leads to the result that the highest infill degree used here, 25%, should be combined with pressing diagonally on the top or vertically on the front of the sample in order to reach maximum recovery, depending on the chosen application. These findings will help to define the ideal orientation of such 3D-printed structures when used in bumpers, orthoses, etc.

Future tests will concentrate on testing higher infill degrees as well as newly designed infill patterns and developing further quantitative evaluation methods. Furthermore, varying the printing parameters, such as printing speed and temperature, will be used to further reduce breaking. The main challenge in this research area is to avoid broken bonds, for which a sophisticated design of the structure is necessary. Here, combining the efforts of materials sciences and construction is necessary. 

In a next step, it is possible to integrate a certain conductivity, e.g., by using conductive PLA, and in this way to integrate heating for recovery, but also for adaptive damping and soft robotics, as recently suggested for nanocellulose nanocomposites [30]. 

## Data Availability

The data created in this study are fully depicted in the article.
